# Topical application of marine briarane-type diterpenes effectively inhibits 12-O-tetradecanoylphorbol-13-acetate-induced inflammation and dermatitis in murine skin

**DOI:** 10.1186/1423-0127-18-94

**Published:** 2011-12-21

**Authors:** Wen-Chi Wei, Sheng-Yen Lin, Yi-Jyun Chen, Chih-Chun Wen, Chiung-Yao Huang, Arulselvan Palanisamy, Ning-Sun Yang, Jyh-Horng Sheu

**Affiliations:** 1 Agricultural Biotechnology Research Center, Academia Sinica, Taiwan; 2 Department of Marine Biotechnology and Resources, National Sun Yat-sen University, Kaohsiung, Taiwan; 3 Graduate Institute of Life Science, National Defense Medical Center, Taipei, Taiwan; 4 Department of Life Science, National Central University, Taoyuan County, Taiwan; 5 Department of Life Science, National Taiwan University, Taipei, Taiwan; 6 Asia-Pacific Ocean Research Center, National Sun Yat-Sen University, Kaohsiung, Taiwan

**Keywords:** Briarane-type diterpene, TPA-induced mouse dermatitis, Dendritic cells, Vascular permeability, Edema

## Abstract

**Background:**

Skin is the largest organ in the body, and is directly exposed to extrinsic assaults. As such, the skin plays a central role in host defense and the cutaneous immune system is able to elicit specific local inflammatory and systemic immune responses against harmful stimuli. 12-O-tetradecanoylphorbol-13-acetate (TPA) can stimulate acute and chronic inflammation and tumor promotion in skin. TPA-induced dermatitis is thus a useful *in vivo *pharmacological platform for drug discovery. In this study, the inhibitory effect of briarane-type diterpenes (BrDs) from marine coral *Briareum excavatum *on TPA-induced dermatitis and dendritic cell (DC) function was explored.

**Methods:**

Evans blue dye exudation was used to determine vascular permeability. H&E-stained skin section was used to determine the formation of edema in mouse abdominal skin. We also used immunohistochemistry staining and western blot assays to evaluate the activation of specific inflammation makers and key mediators of signaling pathway in the mouse skin. Furthermore, mouse bone marrow DCs were used to determine the relationship between the chemical structure of BrDs and their regulation of DC function.

**Results:**

BrD1 remarkably suppressed TPA-induced vascular permeability and edema in skin. At the biochemical level, BrD1 inhibited TPA-induced expression of cyclooxygenase-2, inducible nitric oxide synthase and matrix metalloproteinase-9, the key indicators of cutaneous inflammation. This inhibition was apparently mediated by interference with the Akt/NF-κB-mediated signaling network. BrD1 also inhibited TNF-α and IL-6 expression in LPS-stimulated BMDCs. The 8, 17-epoxide of BrDs played a crucial role in the inhibition of IL-6 expression, and replacement of the C-12 hydroxyl group with longer esters in BrDs gradually decreased this inhibitory activity.

**Conclusions:**

Our results suggest that BrDs warrant further investigation as natural immunomodulatory agents for control of inflammatory skin diseases.

## Background

Skin is the largest organ in the body. As the primary interface between the body and environment, it serves as the first line of defense against microbial pathogens as well as physical and chemical stress or insults [1,2]. The skin does not only serve as a physical and a chemical barrier, but is also an immune-competent organ that elicits effective innate and adaptive immune responses to protect the human body. The cutaneous immune system maintains a balance between restricting excessive inflammation following tissue damage or injury and preserving the ability to rapidly respond to pathogen infection [3]. It is clear that inadequate or misdirected immune response is involved in the pathogenesis of a variety of acquired inflammatory skin disorders [1]. Therefore, systematic investigation of the mechanisms of action of immunomodulatory agents on the skin's immune system is necessary for the development of therapies for skin disorders.

Acute inflammation is the initial immune response to harmful stimuli. Acute inflammation in the skin often involves an increase in the vascular permeability of skin tissues, resulting in an accumulation of fluid at the inflamed site (edema). The release of mediator molecules such as nitric oxide and prostaglandins also elicits vascular permeability, thus permitting the efficient migration of leukocytes, mainly neutrophils, to the inflamed tissue site. Matrix metalloproteinase-9 (MMP-9) has been reported to be a crucial player in such neutrophil migration by degrading some major cellular components of the epidermis and dermis [4]. In addition, it is well-known that secretions of cytokines such as TNF-α, IL-1α and IL-6 by keratinocytes or antigen-specific cells can play a key role in mediating the cutaneous inflammatory response [2,5,6]. These mediators were employed as indicators of skin inflammation in this study.

Several novel approaches have been explored to manage risk factors for skin cancers, tissue damage from UVB exposure, and inflammatory skin disorders [7]. Several phytochemicals and tissue extracts from medicinal plants have been reported to confer immunostimulatory activities and have potential clinical applications [8,9]. Our laboratories previously reported that a small phytochemical from *Lithospermum erythrorhizon *(shikonin) can inhibit the transcriptional activation of human TNF-α promoter *in vivo *in mouse skin [10]. We also showed that caffeic acid suppresses UVB radiation-induced expression of IL-10 and activation of MAPKs in mouse skin tissues [11]. More recently, we demonstrated that ferulic acid, a phenolic phytochemical, can effectively inhibit UVB-induced matrix metalloproteinases in mouse skin via a posttranslational mechanism [7].

Natural products from plants and terrestrial microorganisms have traditionally provided good sources of lead compounds/agents for human medicines. However, due to the biological diversity of the marine environment and the discovery of marine compounds with certain unique structures and pharmacological activities, compounds from marine organisms are expected to be a major source of lead compounds for future generations of pharmaceuticals [12]. A spectrum of different novel marine compounds have been identified, and their bioactivities evaluated for potential pharmaceutical application [13]. Among these compounds, we isolated and identified a group of briarane-type diterpenes (BrDs) from *Briareum excavatum*, a Formosan gorgonian coral [14,15].

In this study, we selected a group of specific BrDs from our chemical library of marine origin for systematic study and showed that BrD1 (excavatolide B) can drastically inhibit 12-O-tetradecanoylphorbol-13-acetate (TPA)-induced acute inflammation in murine skins, as determined by its effect on vascular permeability, edema and key inflammatory mediators. We further determined a structure-activity relationship (SAR) of selected BrDs in terms of their regulation of cytokine expression. The possible molecular mechanisms for the mode of action of these BrDs were also studied.

## Methods

### Reagents

Recombinant cytokines mIL-4 and mGM-CSF were purchased from PeproTech (Rocky Hill, NJ). TPA (12-O-tetradecanoyl-13-phorbol-acetate), Evans blue dye, *N, N*-dimethyl-formamide and LPS (*Escherichia coli *055:B5) were purchased from Sigma-Aldrich (St. Louis, MO). BrDs were prepared from marine soft coral (*Briareum excavatum*) as previously described [14].

### Mice

Female C57BL/6JNarl mice (5-6 weeks old) were purchased from the National Laboratory Animal Breeding and Research Center, Taipei, Taiwan. All mice were maintained in a laminar airflow cabinet in a specific pathogen free (SPF) animal room kept at 24 ± 2°C and 40-70% humidity with a 12 h light/dark cycle under SPF conditions. All facilities were approved by the Institutional Animal Care and Utilization Committee of Academia Sinica, and animal experiments were all conducted according to institutional guidelines.

### Preparation of 12-acyloxyl analogues of BrD1

An appropriate acyl chloride (0.02 mmol) was added to a solution of BrD1 (excavatolide B) (10 mg) in 5 ml of pyridine; the mixture was allowed to stand overnight at room temperature. Four milliliters of water was added to the reaction mixture followed by extraction with EtOAc (5 ml × 3). The EtOAc layers were combined, dried over anhydrous MgSO_4 _and evaporated. The afforded residue was purified by column chromatography on silica gel using EtOAc/hexane (1:8) as eluent to yield analogues of BrD1 (BrD1-5C, BrD1-6C, BrD1-7C and BrD1-10C). Yields varied from 61.6% to 78.3%.

### Measurement of vascular permeability

TPA-induced vascular permeability assay was modified and performed as previously described [16]. Shaved abdominal skins of female C57BL/6JNarl mice were topically treated with vehicle (acetone, 200 μl/site) or TPA (10 nmol in 200 μl acetone/site) for 6 h or treated with TPA for 10 min and subsequently treated for 6 h with the indicated concentrations of BrD1. Abdominal skins of untreated mice were used as the control group. One percent Evans blue dye (100 μl) was injected into mouse tail veins. After 20 min, anatomical appearances of mouse abdomens from the various treatments were photographically recorded. Abdominal skins representative of each test group were removed, turned over and photographed. Evans blue dye extravasated into the skins was extracted by incubation of the skin samples in 99% *N, N*-dimethyl-formamide overnight at 60°C and optical density was measured at 620 nm.

### Generation of mouse bone marrow-derived dendritic cells

For mouse bone marrow-derived DCs (BMDCs), five to six-week-old female C57BL/6JNarl mice were purchased from the National Laboratory Animal Center, Taiwan and kept under SPF conditions. BMDCs were generated from bone marrow cells of C57BL/6 mice as described previously [17]. In brief, bone marrow was isolated from femurs and tibiae which were then flushed with RPMI-1640 medium using a syringe with a 0.45-mm needle on Day 0. Red blood cells in suspension were lysed for 5 min with ACK lysing buffer (150 mM NH_4_Cl, 1.0 mM KHCO_3_, 0.1 mM EDTA). Bone marrow cells were suspended at a density of 1 × 10^7^cells/30 ml in RPMI-1640 containing 10% FBS, 2 mM L-glutamine, 1% of nonessential amino acids, 100 U/mL penicillin and 100 μg/mL streptomycin supplemented with 20 ng/mL of mGM-CSF in 15-cm dishes at 37°C with 5% CO_2_. On day 2, two-thirds of the medium was removed and 30 mL fresh medium with mGM-CSF was added to the cells. On day 5, culture plates were gently swirled and the floating and loosely adherent cells were discarded. Aliquots of 75% culture media were replenished with 20 ng/mL mGM-CSF and 20 ng/mL mIL-4. On day 7, mouse BMDCs (95% pure CD11b and MHCII) were harvested and incubated in RPMI 1640 medium containing 1 mM sodium pyruvate, 0.1 mM nonessential amino acids, 100 U/ml penicillin, 100 μg/ml streptomycin, and supplemented with 10% fetal bovine serum (Invitrogen, Carlsbad, CA) for 24 h at 37°C with or without test chemicals in the presence or absence of LPS (100 ng/ml).

### Measurement of pro-inflammatory cytokines

BMDCs were treated with or without test chemicals in the presence or absence of LPS (100 ng/ml) for 24 h at 37°C. Aliquots of supernatants from DC cultures were assayed for IL-6 and TNF-α using commercial ELISA kits (R&D Systems, Minneapolis, MN) following the manufacturer's recommendations.

### Primer design and RT-PCR

**YES: Yesp **Shaven abdominal skins of female C57BL/6JNarl mice were untreated or topically treated with vehicle (acetone, 200 ml/site) or TPA (10 nmol in 200 ml acetone/site) for 6 h or treated first with TPA for 10 min and then with the indicated concentrations of BrD1 for 6 h. Total RNA was extracted from treated abdominal skins of female mice using TRIzol reagent (Invitrogen Corp., Carlsbad, CA). RT-PCR reactions using AccessQuick RT-PCR system (Promega, Madison, WI) were carried out as described previously [7]. The primers contained the following sequences: mouse MMP-9 sense primer 5'-CTAGTGAGAGACTCTACACGGAG-3', and anti-sense primer 5'-GAGCCACGACCATACAGATACTG-3'; mouse GAPDH sense primer 5'-CATCACTGCCACCCAGAAGACTGTGGA-3', and anti-sense primer 5'-TACTCCTTGGAGGCCATGTAGGCCATG-3'. Gel images were scanned and densitometry analysis of the captured image was performed using Gene Tools software (Syngene, Cambridge, UK).

### Histopathological analysis

Mice were treated or not treated topically on their shaven abdomens with acetone (vehicle only) or TPA (10 nmol in 200 μl acetone/site) for 6 h or treated first with TPA for 10 min, then with the indicated concentrations of BrD1 for 6 h. Mice were killed by cervical dislocation. Abdominal skin tissues were collected, fixed with formalin buffer and embedded in paraffin. Tissue sections (5 mm) were cut and laid onto silanized glass slides and deparaffinized three times with xylene for 5 min each prior to rehydration using a graded alcohol bath. For hematoxyline and eosin staining, the sections were stained with hematoxyline and eosin staining. For immunohistochemical staining, the deparaffinized sections were boiled in 10 mM citrate buffer (pH 6.0) for 10 min for antigen retrieval and rinsed with PBS containing 0.05% Tween-20 buffer for 5 min. The sections were treated with 3% hydrogen peroxide in methanol for 15 min to decrease non-specific binding. They were subsequently washed with blocking solution (PBS containing 1% BSA) for 30 min and then PBST twice for 5 min. All slides were incubated first with 2% goat serum in blocking solution for 30 min, then with a 1:200 dilution of polyclonal iNOS antibody (eBioscience, San Diego, CA) at room temperature for 1 h. The slides were further developed using HPR EnVisionTM system (Dako, Glostrup, Denmark). Subsequently, peroxidase-binding sites were determined by staining with 3,3'-diaminobenzidine tetrahydrochloride (Dako). Eventually, Mayer's hematoxyline was used for counterstaining.

### Western blotting analysis

Shaven abdominal skins of female C57BL/6JNarl mice were not treated or topically treated with vehicle (acetone, 200 μl/site) or TPA (10 nmol in 200 μl acetone/site) at indicated time points or treated first with TPA for 10 min, then with the indicated concentrations of BrD1 at the indicated time points. Abdominal skin samples were collected, homogenated and lysed to prepare total proteins. Protein samples were subsequently resolved by SDS-PAGE using a gradient gel. The resolved proteins were transferred to a PVDF Immobilon-P membrane (Millipore, Bedford, CA.), and the membrane was blocked with 5% non-fat dry milk in PBST buffer (phosphate-buffered saline (PBS) containing 0.1% Tween 20) for 60 minutes at room temperature. The membranes were incubated overnight at 4°C with commercially available antibodies (1:1000 dilutions). Loading of equal amounts of protein was assessed using mouse β-actin. The blots were rinsed three times with PBST buffer for 5 minutes. Washed blots were incubated with HRP-conjugated secondary antibody (1:100,000 dilution), then washed again three times with PBST buffer. The transferred proteins were visualized with an enhanced chemiluminescence (ECL) detection kit (Amersham Pharmacia Biotech, Buckinghamshire, UK). Quantification of bands was performed using Image J software.

### Statistical analysis

Statistical analyses were performed with GraphPad Prism software, version 5. Data are presented as mean ± SD and statistical significance was determined by a one-way ANOVA followed by Tukey multiple comparison tests. Means were considered significantly different if the P value was less than 0.05.

## Results

### Excavatolide B (BrD1) effectively inhibits TPA-induced vascular permeability in mouse skin

**Figure 1:**
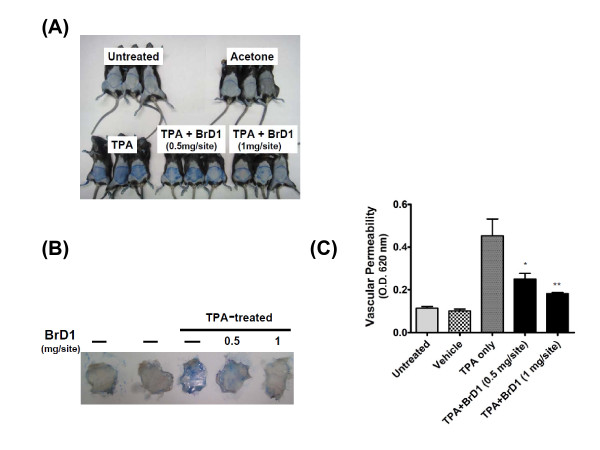
**Briarane-type diterpene 1 (BrD1) inhibits TPA-induced vascular permeability in mouse skin**. Abdominal skins of female C57BL/6 mice were treated topically with TPA (10 nmol) or acetone (vehicle control) for 6 h, or treated with TPA for 10 min and then treated for 6 h with the indicated concentrations of BrD1. One percent Evans blue dye (100 μl) was injected into mouse tail veins for 20 min. (A) Photograph of mouse abdominal skins subjected to various treatments and vascular permeability test. (B) Photographs of the dermal (internal) sides of representative abdominal skins subjected to the above treatment and test. (C) Evans blue extravasation in test skins was determined by assay of optical density at 620 nm. *, *P *< 0.05, and **, *P *< 0.01 versus LPS control. Data are representative of two independent experiments.

Acute inflammatory reactions are known to include changes in vascular permeability, edema and cellular infiltration [18]. In order to evaluate the anti-inflammatory effect of BrD1 on TPA-induced dermatitis in a murine model, we initially evaluated the possible inhibitory effect of BrD1 on TPA-induced vascular permeability. Abdomens of female C57BL/6 mice were treated topically with TPA (10 nmol) or acetone (vehicle control) for 6 h, or treated first with TPA for 10 min and then with the indicated concentrations of BrD1 for 6 h. Mice were injected for 20 min via the tail vein with 100 μl of 1% Evans blue. Consistent with previous reports, TPA strongly increased vascular permeability (Figure 1A, B and 1C). Topical application of BrD1 after TPA treatment significantly inhibited the TPA-induced vascular permeability. The level of inhibition was approximately 58% (0.5 mg/site of BrD1) and 77% (1 mg/site of BrD1) (Figure 1C).

### BrD1 inhibits TPA-induced edema

**Figure 2:**
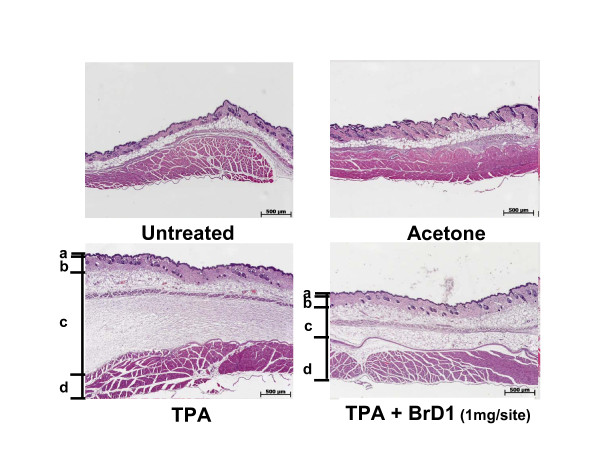
**BrD1 inhibits TPA-induced edema**. Abdominal skins of female C57BL/6 mice were topically treated as described above in Figure 1. Skin biopsies of abdominal skins were collected and stained with hematoxylin and eosin. **a, b, c **and **d **indicate the tissue layers: epidermis, dermis, hypodermis and peritoneum, respectively. Data are representative of two independent experiments.

Since increased vascular permeability is one of factors contributing to the formation of edema, we next evaluated whether BrD1 inhibited formation of edema in TPA-induced inflammation by examining H&E-stained longitudinal sections of skin samples. As seen in Figure 2, topical application of TPA markedly increased the skin thickness, especially in the hypodermal layer as compared with that of untreated or vehicle control groups. Treatment with BrD1 after TPA application substantially decreased the skin thickness, indicating BrD1 can effectively inhibit TPA-induced edema in mouse abdominal skin.

### BrD1 suppresses TPA-induced COX-2 and iNOS expression in mouse skin

**Figure 3:**
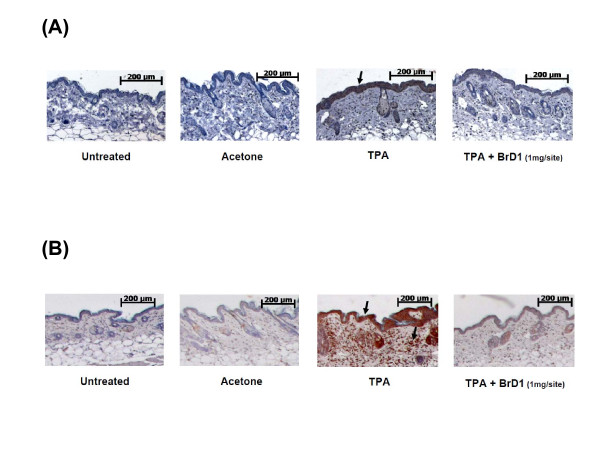
**BrD1 suppresses TPA-induced iNOS expression in mouse skin**. Abdominal skins of female C57BL/6 mice were topically treated as described in Figure 1. Longitudinal tissue sections of abdominal skins were immunostained for COX-2 (A) and iNOS (B) proteins and counter-stained with hematoxylin, as described in Materials and Methods. Positive staining for COX-2 and iNOS are visualized as brownish cells in the dermis and epidermis (arrow). Data are representative of two independent experiments.

Cyclooxygenase-2 (COX-2) and inducible nitric oxide synthase (iNOS) are key mediators of various inflammation and immunity activities. A relatively high level of COX-2 and iNOS expression can be observed in skin during the acute phase of inflammation [19]. To examine whether BrD1 can also inhibit COX-2 and iNOS expression in TPA-inflamed skin, we applied BrD1 topically to TPA-treated skin for 6 h, and then stained it immunohistochemically with anti-COX-2 and anti-iNOS antibodies. As seen in Figure 3A and 3B, TPA treatment stimulated COX-2 expression in the epidermal layer, and TPA treatment strongly stimulated iNOS production in the epidermal and dermal layers as compared with those of the untreated or vehicle control-treated mice. Furthermore, BrD1 treatment reduced the TPA-induced COX-2 expression in the epidermis (Figure 3A) and markedly reduced the TPA-induced iNOS expression in the epidermal and dermal layers of test mouse skin (Figure 3B).

### BrD1 inhibits TPA-induced MMP-9 expression in mouse skin

**Figure 4:**
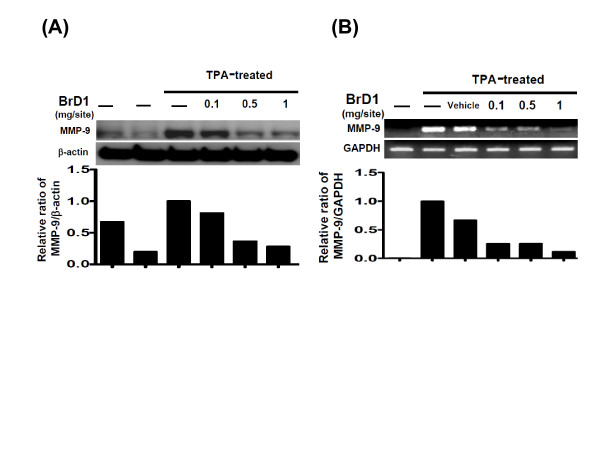
**BrD1 inhibits TPA-induced MMP-9 expression in mouse skin**. (A) Abdominal skins of female C57BL/6 mice were treated topically with TPA (10 nmol) or acetone (vehicle control) for 24 h, or treated with TPA for 10 min and then treated for 24 h with indicated concentrations of BrD1. Skin samples were collected, processed and analyzed for MMP-9 protein expression using western blot analysis. Mouse β-actin was used as a control. (B) Abdominal skins of female C57BL/6 mice were topically treated as described in Figure 1. MMP-9 mRNA expression was determined using RT-PCR assay. The ratio is presented as the value relative to the intensity of TPA-treated control skin. Data are representative of two independent experiments.

Matrix metalloproteinases (MMPs) play a crucial role in many physiological and pathological processes through remodeling extracellular matrix tissues [20]. Matrix metalloproteinase-9 (MMP-9), a gelatinase which has a key role in inflammatory response, is activated in skin during tissue injury [21]. In order to evaluate the anti-inflammatory effect of BrD1 on MMP-9 activation in TPA-inflamed skin, BrD1 was topically applied after TPA treatment for 24 h. As seen in Figure 4A, TPA treatment vigorously stimulated MMP-9 protein expression as compared with untreated or vehicle control groups. On the other hand, BrD1 significantly inhibited TPA-induced MMP-9 protein expression in a dose-dependent manner. Inhibition with BrD1 treatment (1 mg/site/mouse) was up to a maximum 89%. Inhibiton of TPA-induced MMP-9 expression by BrD1 treatment could also be readily observed at the mRNA level. As shown in Figure 4B, TPA strongly stimulated MMP-9 mRNA expression and BrD1 applied topically after TPA treatment also significantly inhibited MMP-9 mRNA expression in a dose-dependent manner. BrD1 treatment (1 mg/site/mouse) inhibited MMP-9 mRNA expression up to a maximum 82%.

### BrD1 inhibits TPA-stimulated NF-κB and Akt activation in mouse skin

**Figure 5:**
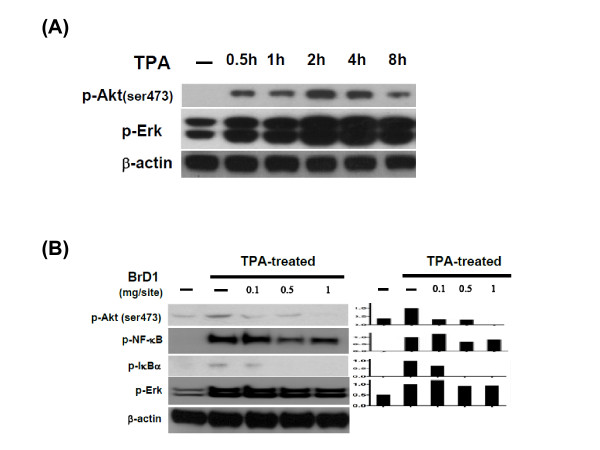
**BrD1 inhibits TPA-induced NF-kB and Akt activation in mouse skin**. (A) Mouse skin was untreated or TPA-treated for indicated time points, and test skin samples were collected and analyzed for phosphorylation levels of Akt and Erk1/2. (B) Abdominal skins of female C57BL/6 mice were treated topically with TPA (10 nmol) or acetone for 2 h or treated with TPA for 10 min and then treated for 2 h with the indicated concentrations of BrD1 for 2 h. Skin samples were collected, processed and analyzed for the phosphorylation levels of Akt, NF-κB, IκBα and Erk1/2 by Western blot analysis. Mouse β-actin was used as a control. The ratio is presented as the value relative to the intensity of TPA-treated control skin. Data are representative of two independent experiments.

The roles of the Akt and Erk signaling pathways in inflammatory activities in mouse skin have been well-demonstrated. We therefore evaluated whether BrD1 could interfere with TPA-induced activation of Akt and Erk in inflamed skin. In order to assess the kinetics of Akt and Erk activation, abdominal skin was stimulated with TPA for 0.5 to 8 h. A high level of phosphorylation of Akt and Erk was observed at 2 h after TPA treatment (Figure 5A). We therefore chose 2 h as the time point at which to assess the BrD1-associated signaling transduction activity in TPA-inflamed skin. The immuno-associated transcription factor NF-κB is a key upstream mediator of iNOS and MMP-9 expressions, and is known to be involved in various cutaneous inflammatory responses. We thus determined the phosphorylation level of Akt, Erk, NF-κB and IκBα in skins subjected to different treatments. TPA treatment strongly stimulated the phosphorylation of Akt, Erk, NF-κB and IκBα (Figure 5B). BrD1 seemed to inhibit TPA-induced Erk phosphorylation only slightly. However, BrD1 treatment effectively inhibited the phoshporylation of NF-κB, and also significantly inhibited the TPA-stimulated phosphorylation of Akt and IκBα. These results indicate that BrD1 can significantly inhibit TPA-stimulated NF-κB and Akt activation in test mouse skin.

### BrD1 inhibits LPS-induced IL-6 and TNF-α expression in mouse bone marrow-derived dendritic cells

**Figure 6:**
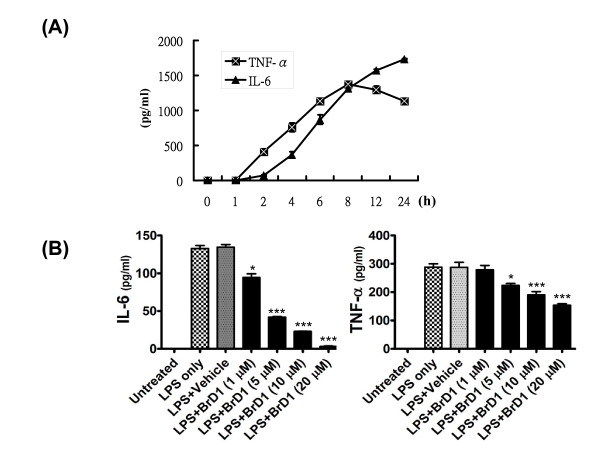
**BrD1 inhibits LPS-induced IL-6 and TNF-α expression in mouse BMDCs**. (A) BMDCs from C57BL/6 mice were treated with LPS (100 ng/ml) for 1 to 24 h. (B) BMDCs from C57BL/6 mice were treated with LPS (100 ng/ml) for 24 h or LPS plus BrD1 at different concentrations for 24 h. Levels of IL-6 and TNF-α proteins in supernatants of conditioned media were analyzed by ELISA. *, *P *< 0.05, and ***, *P *< 0.001 versus LPS control. Data are representative of two independent experiments.

Several studies have reported that the secretion of specific cytokines, including IL-1β, TNF-α and IL-6 by keratinocytes and various immune cells is involved in cutaneous inflammation [2,5,6]. In order to investigate the anti-inflammatory effect of BrD1 on cytokine expression, mouse bone marrow-derived dendritic cells were used to evaluate the inhibitory effect of BrD1 on LPS-induced expressions of TNF-α and IL-6. In our time-course study for expression of TNF-α and IL-6, after LPS stimulation, a high expression of TNF-α and IL-6 was maintained at 24 h (Figure 6A). We therefore chose 24 h as the time point at which to assess the expression of TNF-α and IL-6. BrD1 treatment strongly inhibited IL-6 expression in LPS-stimulated dendritic cells, and inhibited TNF-α expression to a much lesser extent (Figure 6B).

### Structure-activity relationship of BrDs that underlies inhibition of LPS-induced IL-6 expression

**Figure 7:**
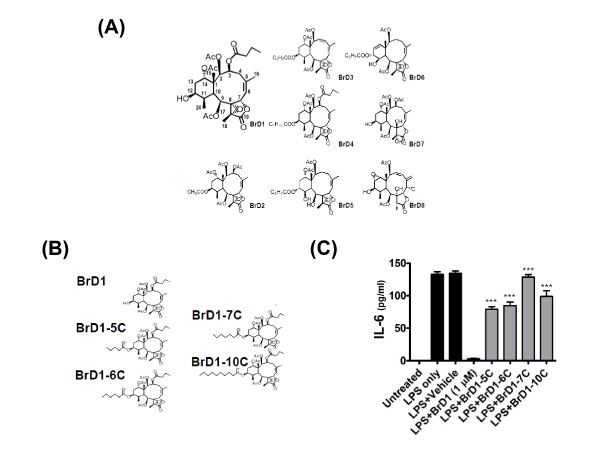
**Structure-activity relationship of briarane-type diterpenes that confer the inhibition of LPS-induced IL-6 expression in mouse BMDCs**. (A) Chemical structures of briarane-type diterpenes **1 **to **8**. The numbers labeling BrD1 indicate the carbon atoms. (B) The chemical structure of BrD1, and its analogs including BrD1-5C, BrD1-6C, BrD1-7C and BrD1-10C. (C) The chemical structure of BrD1, and its analogs including BrD1-5C, BrD1-6C, BrD1-7C and BrD1-10C. Immature DCs were co-treated with LPS and BrDs at 20 μM. The level of IL-6 proteins in supernatants was determined using ELISA. ***, *P *< 0.001 versus LPS+BrD1 treatment. Data are representative of two independent experiments.

In order to explore the relationship between the chemical structures of the BrDs and their inhibition of inflammation, immature DCs were co-treated for 24 h with LPS and BrDs (BrD1-BrD8) at 20 μM. Initially, IL-6 was used as a target to evaluate the inhibitory activity of the BrDs. As seen in Table 1 and Figure 7A, the BrDs exhibited a broad spectrum of inhibition in test mouse DCs. The range of inhibition was between 97.6% (BrD1) and 18.7% (BrD8). Importantly, MTT cell viability assays showed that treatment with BrDs at the test concentration of 20 μM for 24 h had no significant effect on the cytotoxicity of test DCs (data not shown). As shown in Table 1 and Figure 6, BrD1 to BrD6 with 8, 17-epoxides inhibited IL-6 expression more strongly than BrD7 and BrD8 without 8, 17-epoxides indicating that the 8, 17-epoxides of BrDs are crucial for the inhibition of LPS-induced IL-6 expression by BrDs. In addition, BrD5 and BrD6 with α-functional groups at C-12 were found to inhibit IL-6 expression less vigorously than BrD1 to BrD4 without α-functional groups at C-12, suggesting that the α position of the functional group at C-12 may have impaired the inhibitory capacity of these compounds (Table 1 and Figure 7A). Furthermore, replacement of the C-12 hydroxyl group in BrD2 to BrD4 with longer ester chains gradually decreased the level of inhibition of IL-6 expression by these BrDs, suggesting that the steric hindrance of longer acyloxyl groups can inhibit the capacity of the briarane-type diterpenes to inhibit of LPS-induced IL-6 expression (Table 1 and Figure 7A).

**Table 1:** Inhibitory effect of briarane-type diterpenes from marine coral *Briareum excavatumon *on LPS-induced IL-6 in mouse BMDCs.

IL-6 (% of LPS control)		
Code	Name (20 μM)	% (Mean ± SD)
BrD1	Excavatolide B	2.4 ± 0.93

BrD2	Excavatolide K	22.5 ± 4.5

BrD3	Excavatolide F	26.2 ± 4.0

BrD4	Briaexcavatolide R	34.7 ± 8.6

BrD5	Excavatolide Z	37.0 ± 9.6

BrD6	Briaexcavatolide B	56.6 ± 2.1

BrD7	Briaexcavatolide K	61.3 ± 9.5

BrD8	Briaexcavatolide H	81.3 ± 1.9

IL-6 in supernatants of diterpenes BrD1 to BrD8 were determined by using a standard sandwich ELISA. Results are expressed as a percentage of LPS-induced IL-6 expression.

### Steric hindrance of 12-acyloxyl substituents reduces the inhibitory bioactivity of briarane-type diterpenes

Our results suggest that steric hindrance of the 12-acyloxyl substituents of BrDs decreases their inhibitory activity. In order to confirm this SAR, we semi-synthesized several analogues of BrD1 by replacing the hydroxyl group at C-12 with longer acyloxyl groups (C_5_, C_6_, C_7 _and C_10_) (Figure 7B). We then examined the inhibitory effect of BrD1 and these BrD1 analogues (BrD1-5C, BrD1-6C, BrD1-7C and BrD1-10C) on cytokine secretion activity in LPS-stimulated mature DCs. BrD1 significantly inhibited the expression of cytokine IL-6 in LPS-stimulated, mature DCs (Figure 7C). This inhibitory activity gradually decreased when the hydroxyl group at C-12 was replaced by longer acyloxyl groups (C_5 _to C_10_), confirming that steric hindrance of 12-acyloxyl substituents can effectively suppress the capacity of BrDs to inhibit LPS-induced IL-6 expression. These results thus strongly suggest that the 8, 17-epoxide and 12-hydroxyl groups of BrD1 are instrumental in conferring the BrD-mediated inhibitory activity in mouse bone marrow-derived dendritic cells.

## Discussion

The immune response in skin tissues is systemic and sophisticated. A variety of vaccination strategies are administered through the skin to produce antigen-specific immune responses and protect the host [22-25]. The skin is also a useful organ for drug delivery, for example, certain pharmaceuticals can be delivered via the use of a transdermal patch. On the other hand, excessive inflammation or inadequate immune response in skin can lead to various cutaneous diseases such as allergic reactions, psoriasis and even skin cancers. Our laboratories have previously demonstrated that a number of phytochemicals derived from medicinal plants or fruits exhibit strong anti-inflammatory effects either on TPA-induced inflammatory mediators or UVB radiation-induced cytokine and MMP expression in mouse skin tissues [7,11,26]. In the present study, we showed that a group of marine briarane-type diterpenes, particularly BrD1, can strongly suppress TPA-induced inflammation and dermatitis in a mouse skin model. In combination with our previous studies [7,10,11,26], the current study leads us to suggest that a specific combination of several skin model/system test sets [27] may warrant a systematic evaluation for use as the first *in vivo *platform for drug screening or efficacy verifications.

Epidermis tissue contains keratinocytes, Langerhans cells and other cells types. When the epidermis is exposed to 12-O-tetradecanoylphorbol-13-acetate (TPA), keratinocytes and Langerhans cells are stimulated through activation of protein kinase C (PKC) resulting in activation of PI3K/Akt, Erk and NF-κB signaling transduction pathway [28-30]. This activation of Akt and NF-κB activity via TPA stimulation was faithfully observed in the present investigation (Figure 5A), providing a solid baseline from which to investigate the effect of BrD1. Akt and NF-κB play crucial roles in the activation of key inflammatory enzymes such as inducible nitric oxide synthase (iNOS) and cyclooxygenase-2 (COX-2) [31-33]. Interestingly, our results showed that the expression of COX-2 and iNOS was detected mainly in the epidermis after TPA stimulation, indicating that COX-2 and iNOS were mainly released from epidermal cells (likely mainly from keratinocytes) (Figure 3A and 3B). In addition, we also observed considerable sporadic expression of iNOS in the dermal layer after TPA treatment, and BrD1 strongly inhibited this iNOS expression in both the epidermal and dermal layers (Figure 3B). The specific cell types in these two tissue layers that are responsive to BrD1 inhibition effect need to be further investigated. The major dermis cell types are fibroblasts, macrophages and fabric basophils (mast cells). During inflammation, immune cells, mainly neutrophils, can effectively infiltrate into the dermal layer. Keratinocytes, mast cells and infiltrated neutrophils in skin have been reported to secrete MMP-9 for tissue remodeling in response to inflammation [34-36]. MMP-9 has further been reported to be directly mechanistically involved in the increases of vascular permeability [37-39]. Our results (Figure 4A and 4B) show that BrD1 can effectively inhibit TPA-induced MMP-9 expression. We suggest that this may be partly due to the inhibitory effect of BrD1 on vascular permeability and edema. Together, our present results suggest that BrD1 may suppress the inflammatory responses mediated by different types of cells present in different tissue layers of the skin. Our data (Figure 5A and 5B) also indicate that the molecular mechanisms responsible for the anti-inflammatory effects of BrD1 may involve the Akt and NF-κB signaling transduction pathway.

The pro-inflammatory cytokines TNF-α and IL-6, released by dendritic cells, are key mediators of inflammatory and immune responses. It is known that expression of these cytokines is controlled by TLR4-stimulated activation of NF-κB via regulation of the transcriptional activities of these cytokines in monocytes and dendritic cells. The results from this study show that BrD1 can inhibit the activation of NF-κB in TPA-induced mouse dermatitis (Figure 5B) and in LPS-stimulated mouse BMDCs (data not shown). However, BrD1 exhibited a differential inhibitory effect on TNF-α and IL-6 in LPS-induced mouse BMDCs. As seen in Figure 6B, BrD1 strongly inhibited LPS-induced IL-6 expression but only partially inhibited LPS-induced TNF-α expression. In order to distinguish between TNF-α and IL-6 inhibition, a time course study of LPS-induced TNF-α and IL-6 expression was performed. After LPS stimulation, a relatively high level of expression of TNF-α was detected between 1 h and 2 h post-treatment, and an elevated level of IL-6 expression was detected between 2 h and 4 h post-treatment (Figure 6A). Our previous findings and those of others have shown evidence of post-transcriptional regulation of TNF-α pre-mRNA in resting T-cells, B-cells and monocytes [40-42]. LPS-induced mRNA splicing can lead to massive and rapid expression of TNF-α mRNA in monocytes [42]. The differential effect on expression of TNF-α and IL-6 in LPS-activated BMDCs seen in this study may have implications for the future use of BrD1 in pharmaceutical applications. The detailed molecular mechanisms responsible for this differential effect warrant further investigation.

## Conclusions

In summary, we conclude that marine briarane-type diterpene (BrD1) can not only effectively suppress TPA-induced vascular permeability and edema, but can also decrease the expression of COX-2, iNOS and MMP-9, and reduce the activation of NF-κB and Akt in test mouse skin tissues. In addition, BrD1 can also strongly inhibit IL-6 expression in LPS-stimulated BMDCs, a key immunoregulatory cell type. The 8, 17-epoxide of the BrDs apparently contribute stereochemically to the inhibition of IL-6 expression, and steric hindrance of the 12-acyloxyl substituents was found to effectively reduce the inhibitory bioactivity of BrD1. In view of the unique structure and specific active functional group of BrD1, we suggest this marine natural product should be further evaluated for development as an immunotherapeutic agent for control of inflammation and skin diseases, and for other healthcare applications.

## Competing interests

The authors declare that they have no competing interests.

## Authors' contributions

Wen-Chi Wei initiated the study, designed and performed the experiments, analyzed data, and wrote the manuscript; Sheng-Yen Lin and Yi-Jyun Chen designed the research, analyzed data, and wrote the manuscript; Chih-Chun Wen and Arulselvan Palanisamy performed some tests and analyzed data; Chiung-Yao Huang contributed vital new reagents; Ning-Sun Yang and Jyh-Horng Sheu established strategies and approaches, designed the research and wrote the manuscript. All authors read and approved the final manuscript.
